# Politics of COVID-19 vaccination in Japan: how governing incumbents’ representation affected regional rollout variation

**DOI:** 10.1186/s12889-023-15376-6

**Published:** 2023-03-17

**Authors:** M. Kikuchi, S. Ishihara, M. Kohno

**Affiliations:** 1 Department of Political Science, Washington University in St. Louis, MO Saint Louis, USA; 2 Department of Global Political Economy, Waseda University, Tokyo, Japan; 3 Faculty of Political Science and Economics, Waseda University, Tokyo, Japan

**Keywords:** COVID-19, Vaccination, Japan, Political representation, Regional variance, Democracy, Electoral incentives

## Abstract

**Background:**

Despite initial delay, Japan’s COVID-19 vaccination accelerated remarkably from May to September 2021 under the leadership of Prime Minister Yoshihide Suga. His “campaign” for vaccination, however, did not yield uniform results nationwide.

**Methods:**

To highlight political determinants for the regional variation, we employ ordinary least squares regression analyses to investigate how the share/presence of incumbent politicians belonging to the governing parties, the Liberal Democratic Party and Komei Party, influenced the varying progress of rollouts across prefectures as well as across cities/towns/villages. The data on the vaccination rate for all 47 prefectures was obtained from Government Chief Information Officer (CIO)’s Portal, Japan (GCPJ) approximately one month prior to the anticipated general election, the national election for the more important House of Representatives of Japan’s bicameral parliament (Diet). The data for lower administrative units, though its availability was limited to only three prefectures, was obtained from the respective governments of Kagawa and Ehime and from a local newspaper in Gifu.

**Results:**

The findings reveal that at both prefectural and sub-prefectural administrative levels, the share/presence of the governing parties’ representation in the national parliament had a positive and statistically significant effect on the region’s vaccination progress, after controlling for the local proliferation of COVID-19 and demographic characteristics.

**Conclusion:**

Our findings contribute insights into the understudied area of the contemporary COVID-19 health environment, namely how the political dynamics of democracy affect the pattern of vaccine dissemination in Japan.

**Trial registration:**

: Not applicable.

**Supplementary Information:**

The online version contains supplementary material available at 10.1186/s12889-023-15376-6.

## Background

Ever since COVID-19 broke out, vaccination has been placed at the center of governmental measures in combating the pandemic around the world. Already a large body of studies has been undertaken by researchers, in both social science and medicine, to explore demographic, psychological and socio-economic factors that may influence individual tendency toward accepting and/or hesitating vaccination [1–8]. While such factors related to ordinary citizens’ attitudes toward vaccination have thus been widely investigated, relatively fewer research has probed how the incentives of political elites, inherent especially in the electoral dynamics of democracy, affect the pattern of vaccine dissemination in developed countries.

We seek to contribute insights into this understudied area of the contemporary COVID-19 health environment, highlighting political determinants of regional vaccination variation in Japan. As described below in some details, Japan accelerated the process of vaccination from May to September 2021, under the leadership of Prime Minister Yoshihide Suga. For Suga himself, as well as for incumbent members of the two parties in the governing coalition, the Liberal Democratic Party (LDP) and Komei Party, it was imminently critical to improve the vaccination rate, because the general election for the House of Representatives, the more important lower house of Japan’s bicameral parliament (Diet), was anticipated to be held at some point in October due to the constitutional term limitation. How, in light of this electoral cycle, did the share/presence of incumbent politicians belonging to the two governing parties influence vaccine rollouts that varied regionally? We show based on cross-sectional regression analyses that, at both prefectural and sub-prefectural administrative levels, the share/presence of the governing parties’ representation in the national parliament had a positive and statistically significant effect on the region’s vaccination progress, after controlling for the local proliferation of COVID-19 and demographic characteristics.

Japan began COVID-19 vaccination as late as February 2021, roughly two months after many other countries had started theirs. By then, the number of cases of new infection and death related to the pandemic had been on the rise again, and the state of emergency had been declared for the second time in Tokyo and elsewhere. The vaccination priority was given at first to healthcare workers, and then to those aged 65 or older. Mass vaccination rollouts, however, lagged way behind. As of May 21, for example, the proportion of the population that had received at least one shot was reported to be about 4%, the lowest amongst developed countries at the time [9]. This initial delay was due to a number of factors, including holdups in vaccine importation and Japanese bureaucrats’ adherence to the routine regulations, especially their insistence on domestic trials before approving imported medicines. The law that technically permitted only doctors to administer the vaccine jab constituted a hurdle as well; it was not until April and May that this rule was sequentially relaxed to allow others in medical professions, such as dentists and clinical laboratory technicians, to participate in COVID-19 vaccination procedure [10].

As ordinary citizens were anxiously waiting for their turns of the first shots, it was reported widely in the news media that politicians in the governing coalition, particularly Prime Minister Yoshihide Suga himself, became frustrated with the slow progress of inoculation [11, 12]. Since taking the office in the previous fall, Suga had been emphasizing the primacy of vaccination in the governmental measures against the pandemic. As Suga had pledged repeatedly, at press conferences and in parliamentary debates, that he would secure enough vaccines for the entire population by the end of June 2021, his accountability was at stake [13]. Also, as the summer approached, there was mounting pressure from the public, majority of whom opposed to holding the Tokyo Olympic/Paralympic Games, fearing further expansion of infection. Suga, however, was determined not to cancel or re-postpone the world event, because it had already been rescheduled from the originally planned summer of 2020 [14, 15]. As a result, his popularity plunged and those who “approved” the government were outnumbered by those who “disapproved” in every major public opinion poll [16].

What further complicated Suga’s predicament were two critical items on his political calendar. First, because of the constitutional term limitation, the House of Representatives had to be dissolved some time before the end of October 2021. Suga, as the leader of the governing LDP, had to look for a window of opportunities, hoping to call a general election on his own term for political advantages; that window would not open, he knew, without a drastic improvement in the vaccination rate and peaking-out of the ongoing wave of the pandemic. Second, Suga’s interim term as LDP President was to end even earlier in September. Not having an organized faction of his own within the party, nothing other than a victory in the general election would secure the chance for him to remain in the position.

It was in this context that Suga took new initiatives, as he expressed his personal determination at his May 7 press conference: “I myself will stand at the fore and achieve an acceleration in our vaccinations” [17]. Suga was tactful in circumventing bureaucratic red tape by publicly, and thus preemptively, announcing his timetable and target. For example, he set the timetable for completing the first vaccine shots for those aged 65 or older by the end of July. He also vowed that the government target vaccinating 1 million people a day [11, 18]. Furthermore, Suga himself took an extraordinary step of requesting the Minister of Defense that Japan’s Self Defense Force open large-scale vaccination centers in two of the big cities, Tokyo and Osaka [12]. These centers were opened in late May, initially to complete prioritized inoculations, but subsequently the eligibility criteria for vaccination were expanded to include anybody between the ages of 18 and 64. In addition, even as the mass rollout seemed to have finally come to be in full operation, Suga did not forget to update his timetable and target so as to re-tighten his reins on local governments and bureaucracy; in a parliamentary debate in early June, he announced that the government would aim to vaccinate all residents in Japan who wish to receive a shot by the end of November [19].

Suga’s resolve paid off and his directives certainly resulted in a significant acceleration of mass rollout in Japan. Before his full-fledged “campaign” started, there was a significant gap in the vaccination rate between Japan and other countries. However, Japan by September caught up with and even surpassed some of them in terms of the proportion of the population that had been vaccinated at least once (see Figure A1 in Online Appendix). Suga did not shy away from claiming the credit of this turnaround, boasting that the target of vaccinating 1 million people a day was accomplished in mid-June, much earlier than he himself had expected [20]. Indeed, the weekly average of daily vaccinations approached as high as 2 million a day in August (see Figure A2 in Online Appendix), an outcome hardly anybody within the bureaucracy or even within his own party had ever thought possible.

Setting aside Suga’s own credit-claiming, it is far from unambiguous that Japan’s record was truly noteworthy. After all, compared with the two-month setback at the start, it took more than three months, from May through August as noted above, for Japan’s vaccination rate to be at par with other countries’ scores. Furthermore, even if Japan’s “catching-up” can be gauged as success, it was not a success story unique to Japan. For example, as Japan was catching up, comparable progress was also being made in the neighboring country, South Korea (see Figure A1 in Online Appendix).

**Fig. 1:**
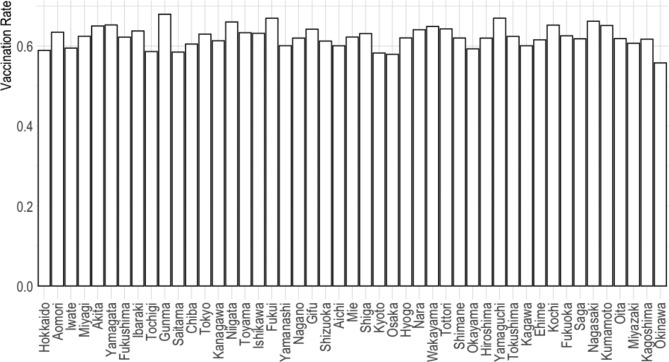
Share of people who received the first doses of COVID-19 vaccine by prefecture (as of September 25, 2021) Data Source: *NHK (Nippon Hoso Kyokai, Japan’s public broadcasting corporation). Special Website: New coronavirus* [21].

What was beyond ambiguity, however, was that the dogged vaccination campaign under Suga’s leadership did not yield uniform progress nationwide. As shown in Fig. 1, the rate of vaccination, as measured as the proportion of those who received the first shots, varied quite substantially across the 47 prefectures, even as late as September 25, the highest being Gunma (67.93%) and the lowest being Okinawa (55.75%). Though not reported here, the rate of the second shot also varied, obviously because of its contingency on, and hence the high correlation with, the rate of the first shot.

The observed regional variation could not have simply reflected factors such as different degrees of concentration of those aged 65 or older. By then, most, if not all, who needed or strongly desired vaccination as early as possible had already received at least their first shots. To explain this variation, we argue that political factors must be taken into account, especially given that the general election, the most important national election, was about to be held at some point in the near future. As described earlier, the political fortune of Prime Minister Suga himself hinged greatly on the outcome of this election. As well, for the individual incumbents who belong to the governing parties, the progress of vaccination in their own constituencies must have been major concerns in calculating reelection chances.

In light of the background summarized in this section, Japan provides an excellent vehicle for a case study which contributes insights into the understudied area of contemporary COVID-19 health environment, namely, how the political dynamics of democracy affect the pattern of vaccine dissemination in developed countries.

In the next section, we outline our methods, detailing the study design, data, variables and statistical models employed. The section on estimation results follows, and the subsequent section discusses implications of our findings. The last section presents the conclusion of the study. A package of supplementary information is provided in online appendix.

## Methods

### Study design

This study employs a cross-sectional design to investigate the relationships between the share/presence of incumbent politicians belonging to the two parties in the governing coalition and the progress of vaccine rollouts in the fall of 2021 in Japan. A cross-sectional design is employed because the data on prefectures and cities/towns/villages within prefectures are regarded to have been collected at a single point in time. As we are to reveal political determinants of cross-sectional variance, regression analyses are conducted at two levels of Japan’s local government: prefectures (Analysis I) and cities/towns/villages within prefectures (Analysis II).

### Data sources and measurement of variables

Our outcome variable is the vaccination rate, and our main independent variable is the governing parties’ representation. For Analysis I, the vaccination rate for all 47 prefectures is calculated based on the data obtained from Government Chief Information Officer (CIO)’s Portal, Japan (GCPJ) [22], as of September 25, 2021, approximately one month prior to the anticipated general election (which was eventually held on October 31). At this official site, “the total number of vaccine doses administered to date” in each prefecture is available with regard to both first and second shots. We focus on the first shot and calculate the rate of vaccination for each prefecture by dividing the number by the (prefecture’s) population based on Japan’s Basic Resident Registrar, Jyumin-Kihon-Daicho [23]. We divide not by the population eligible for vaccines but rather by the total population because, as explained below, the exact demographic variable for both eligible and non-eligible for vaccines, i.e., the cohorts aged 11 or younger and aged older than 11, are not available; we instead control for this group using a proxy variable.

As we are concerned that the number of prefectures is rather small for a multivariate quantitative inquiry, we also collect data for lower administrative units so as to replicate the analysis (Analysis II). The data availability, however, is limited to only three prefectures, Ehime, Gifu, and Kagawa, and the definitions of vaccination rates reported in these prefectures are not identical, requiring extra caution for the analysis and interpretation. Specifically, as for Ehime and Kagawa, the data of vaccination rate for all their cities/towns/villages is available at the official sites of the respective prefectural governments, as of September 10 for Kagawa [24] and September 14 for Ehime [25]. In these two prefectures, the rate of vaccinations is calculated after excluding those aged 11 or younger, the segment of the population who were not entitled for vaccination. The data for Gifu is obtained from a local newspaper, Gifu-Shimbun, as of September 9. (This data, since it is no longer freely accessible, will be provided to anyone interested upon request.) [26] According to this data source available for Gifu, the rate of vaccinations is calculated by excluding not only those aged 11 or younger, but also those aged 65 or older who were prioritized in vaccination. The number of cities, towns and villages combined for these three prefectures totals 79.

Our main independent variable of interest, the governing parties’ representation, is measured by the share/presence of politicians affiliated with the LDP and Komei Party, because these two parties compose Japan’s coalition government at the national level. The estimated sign of this main variable is expected to be positive as the incumbent governing parties’ politicians had an incentive to distribute vaccines to gain electoral advantage. Profiles of incumbent politicians in the House of Representatives, as they all had run as candidates in the previous election, are available at various sites of news organizations, though some of these politicians have changed their partisan affiliations after winning seats. For consistency, we rely on an unofficial, but widely used data source called Kokkai Giin Hakusho, which systematically collects and updates the personal information of all past and present Diet members [27]. Japan’s House of Representatives consists of two tiers of politicians, one elected from local constituencies via single-seat competitions and the other from more broadly categorized “regions” via proportional representation contests. We focus exclusively on the former group of incumbent politicians whose constituencies are demarcated on the prefectural basis. We code a member 1 if the politician belongs to either the LDP or Komei Party, and 0 if otherwise. Based on this codification, we prepare two sets of explanatory variables. For Analysis I, we straightforwardly calculate the “share of the governing parties’ representation in Diet,” by adding up the number of the elected LDP and Komei members per prefecture and then dividing it by the total number of the House seats assigned to each prefecture. For Analysis II, the procedure is not so straightforward, because the electoral districts do not necessarily correspond to the boundaries of lower administrative units. For our purpose, we create a dummy variable, coded 1 if the representative elected from the given city/town/village belonged to either the LDP or Komei Party, and 0 if otherwise. In cases where the administrative unit includes more than one electoral district, we code 1 if at least one LDP or Komei representative was present, and 0 if otherwise.

For Analysis I, in addition to the share of governing parties’ representations in the national parliament, we also calculate the share of governing parties’ representations in the prefectural assemblies. Partisan profiles of incumbents in these assemblies are available at another widely used site called Senkyo Dotto Com [28]. We follow the procedures above to prepare the variable for local representations. We do not pursue preparing a variable that measures the presence/absence of LDP and Komei politicians in city/town/village assemblies for Analysis II, because the partisan landscape at this lowest administrative level, with the presence of many nonpartisan independents and affiliates with local parties, hardly mirrors that of the national level.

### Other control variables

For control, we use several variables, including basic demographic variables and variables that measure the local proliferation of COVID-19, derived from publicly available sources. In addition, we are able to collect data on the number of doctors in each prefecture for Analysis I.

Specifically, demographic variables are included because we need to capture at least both the distribution of the older generation of local residents and that of the younger generation. The expected sign of estimation for the former is positive because the over-65 population was given vaccination priority and that for the latter is negative because the younger generation was not yet entitled for vaccination. For these variables, we rely on Japan’s Basic Resident Registrar [23], which contains population data by different age categories for prefectures and sub-prefectural units. As for the prioritized cohort, we are able to measure the percentages of the local population aged 65 or older; the government formally prioritizes the vaccination for those aged 65 or older. As for those without vaccine entitlement, we are unable to capture this particular cohort, i.e., those aged 11 or younger, because the Registrar divides age categorization by every five years. We therefore calculate the percentages of those aged 9 or younger as a proxy. For Analysis I, the measures of both the prioritized and non-entitled cohorts are included. For Analysis II, the measure of the prioritized cohort is included but that of the non-entitled cohort is not included because the original data sources exclude the younger population group in calculating the vaccination rate.

As the rate of vaccination is likely to be contingent on COVID-19 proliferation, we also need to control for the effect of virus proliferation itself. Specifically, we calculate the rate of infection and the rate of death, as the total number of COVID-19 infections and that of death related to COVID-19 divided by the region’s population, respectively. The expected signs for these variables can be both positive and negative. Positive, if the proliferation is assumed to have an effect of convincing people to get vaccinated sooner. Negative, on the other hand, if a rise in COVID-19 spread implies that those who died due to the infection will no longer be available or those who were infected will lose motivation to receive vaccines, hence leading to a fall in the number of people who would have received the vaccines. For Analysis I, the data on infection and death in each prefecture is drawn from the site of NHK (Nippon Hoso Kyokai, Japan’s public broadcasting corporation) [21]. For Analysis II, the infection data at the sub-prefectural level is collected from the official site of each prefectural government of Ehime [29], Gifu [30], and Kagawa [31]. The data for death counts related to COVID-19 for lower administrative units is not available.

Finally, we need to control for the administrative capacity of vaccination rollouts, given that such capacity imposes the most basic constraint on the opportunities available for vaccination on a daily basis. For this purpose, we collect the data for the number of doctors in each prefecture from the Ministry of Health, Labor and Welfare [32] for Analysis I. The expected sign of estimation for this variable is positive because vaccination rollouts require vaccination provision capacity. The number of nurses is also available from the same data source but is highly correlated with the elderly population; we thus present our analysis that includes the variable of nurses as a potential confounder in Appendix D merely as a robustness check. The data for the number of doctors for the sub-prefectural units for Analysis II is not available.

The data derivation dates and data sources for the variables used are summarized in Table 1.

**Table 1:** Summary of variables

Variable	Definition	Data derivation as of	Data source
*Prefectural Level*			
Vaccination Rate	Rate of the first vaccination over the number of population	Sep. 25, 2021	Government CIO’s Portal, Japan (GCPJ)
% of those aged 65 or older	Proportion of population aged 65 and over	Dec. 31, 2020	Japan’s Basic Resident Registrar
% of those aged 9 or younger	Proportion of population aged 9 or below	Dec. 31, 2020	Japan’s Basic Resident Registrar
Share in National Diet	Share of the governing parties’ members in national diet	Sep. 25, 2021	Kokkai Giin Hakusho
Share in Prefectural Assembly	Share of the governing parties’ members in prefectural assembly	Sep. 25, 2021	Senkyo Dotto Com
Infection Rate	Number of cumulative infections per 10,000	Sep. 25, 2021	NHK
Death Rate	Number of cumulative deaths per 10,000	Sep. 25, 2021	NHK
# of doctors (per 100,000)	Number of doctors per 100,000	Dec. 31, 2018	Ministry of Health, Labor and Welfare
*Cities/Towns/Villages Level*			
Vaccination Rate	Rate of the first vaccination among those aged 12 and over (Ehime and Kagawa) and aged between 12 and 64 (Gifu)	Sep. 14, 2021 (Ehime) Sep. 10, 2021 (Kagawa) Sep. 9, 2021 (Gifu)	Ehime Prefecture (Ehime), Kagawa Prefecture (Kagawa), Gifu Shimbun (Gifu)
% those aged 65 or older	Proportion of population aged 65 and over	Dec. 31, 2020	Japan’s Basic Resident Registrar
Presence in National Diet	1 if a representative belongs to the governing party and 0 if otherwise	Aug. 31, 2021	Kokkai Giin Hakusho
Infection Rate	Number of cumulative infections per 10,000	Sep. 13, 2021 (Ehime) Sep. 9, 2021 (Kagawa) Sep. 8, 2021 (Gifu)	Ehime Prefecture (Ehime), Kagawa Prefecture (Kagawa), Gifu Prefecture (Gifu)

### Statistical analysis

We investigate the association between the share/presence of incumbent politicians belonging to the ruling coalition and the vaccination rate using the ordinary least squares (OLS) model. We adopt the standard OLS method in our estimations because our analysis attempts to capture the relationship between the politicians’ share/presence and vaccination rate, a continuous variable, at a single observation point. Concretely, we run seven models for the prefectural level (Analysis I) and two for the sub-administrative level (Analysis II) with and without control variables. In order for OLS to be valid, the assumption of linear relationship must hold. According to our preliminary analysis, this assumption holds, as the inspection of residuals and fitted values, and Variance Inflation Factor (VIF) altogether confirms the absence of heteroskedasticity and multicollinearity, as well as the appropriateness of the presumed linear functional form (See Online Appendix C). For Analysis II, which focuses on sub-administrative units within the prefectures, we include prefectural fixed effects; it is appropriate to include them especially because of different operationalizations of vaccination rates.

## Results

Table 2 summarizes the estimation results for the analysis of cross-prefectural variance (Analysis I). According to Model 1 which only includes the two basic demographic variables, the proportion of those aged 65 or older has a positive association with the rate of vaccination. Upon including our key explanatory variables of governing parties’ representations, however, the effect of this demographic variable can no longer be confirmed. Throughout the results presented in this table, the two demographic variables are estimated to be in the expected directions respectively (that is, positive for the proportion of those aged 65 or older and negative for that aged 9 or younger) but both variables remain statistically insignificant.

**Table 2:** Regression analysis I: prefectural level

Variable	Model 1	Model 2	Model 3	Model 4	Model 5	Model 6	Model 7
*Demographics*							
% of those aged 65 or older	0.340 ** (0.145)	0.051 (0.233)	0.123 (0.184)	0.061 (0.233)	0.015 (0.237)	0.065 (0.190)	0.022 (0.238)
% of those aged 9 or younger	-0.349 (0.584)	-0.303 (0.590)	-0.568 (0.614)	-0.513 (0.633)	-0.685 (0.615)	-0.912 (0.630)	-0.858 (0.660)
*Governing Parties’ Representation*							
Share in National Diet		0.029** (0.014)	0.031** (0.013)	0.030** (0.014)			
Share in Prefectural Assembly					0.050 (0.031)	0.052* (0.030)	0.049 (0.032)
*COVID-19 Rates (per 10,000)*							
Infection Rate		-0.014 (0.01)		-0.006 (0.013)	-0.011 (0.011)		-0.004 (0.014)
Death Rate			-1.034 (0.618)	-0.785 (0.846)		-0.823 (0.659)	-0.653 (0.868)
# of doctors (per 100,000)		0.003 (0.009)	0.003 (0.009)	0.004 (0.009)	0.005 (0.009)	0.005 (0.009)	0.006 (0.009)
Constant	54.684*** (8.012)	61.150*** (9.932)	60.601*** (9.174)	62.259*** (10.021)	63.442*** (10.019)	63.293*** (9.281)	64.487*** (10.167)
Observations	47	47	47	47	47	47	47
Adjusted R^2^	0.184	0.269	0.281	0.266	0.235	0.244	0.227
Residual Std Error	2.383 (df = 44)	2.256 (df = 41)	2.238 (df = 41)	2.260 (df = 40)	2.308 (df = 41)	2.294 (df = 41)	2.32 (df = 40)
F Static	6.185*** (df = 2; 44)	4.379*** (df = 5; 41)	4.590*** (df = 5; 41)	3.781*** (df = 6; 40)	3.825*** (df = 5; 41)	3.967*** (df = 5; 41)	3.248** (df = 6; 40)

Notes: Standard errors are in parenthesis. *p < 0.1, **p < 0.05, ***p < 0.01

Between the two political variables, the estimated effect of the governing parties’ representation of the national parliament is far more consistent in affecting the outcome variable than that of the prefectural assemblies. Throughout Models 2, 3 and 4, the effect of the share of the governing parties’ representation in Diet is estimated to be positive and statistically significant at 5% level. With regard to the share of the governing parties’ representation in prefectural assemblies, its effect is estimated to be statistically significant only for Model 6, at the 10% significance level. Comparisons of Adjusted R^2^ and F-Statistics also confirm that, generally, the set of models that include the variable for national representation better perform than the models that include the variable for local representation. These results suggest that, at least at the time when this study was conducted, namely at the time when the general election was anticipated to be held in the near future, the number of incumbent politicians belonging to the governing LDP and Komei Party in Diet was associated with the varying progress of rollouts across prefectures.

Turning to other variables, Table 2 indicates that the local spread of the pandemic itself does not seem to have promoted, nor impeded, the progress of mass vaccination rollouts that varied across prefectures: neither the variable for the rate of infection nor that for the rate of death related to COVID-19 is estimated to be consequential in determining the cross-prefectural variation in any of the models analyzed.

According to Table 2, the variable that measures the administrative capacity of vaccination, i.e., the number of doctors who could have been mobilized for rollouts, is inconsequential as well.

Reviewing the results overall, then, the impact of the governing parties’ share in the lower house representation clearly stands out. None of the other explanatory or control variables included performs as powerfully and consistently. Can these results be replicated at the level of lower administrative units as well?

Let us now turn to Table 3, a summary of OLS estimation results for the variance of mass vaccination rollouts across cities, towns and villages within the three prefectures where we were able to obtain data (Analysis II). Note though, for the reasons explained earlier, some of the variables included in the prefectural level analysis are not included; furthermore, we must proceed with caution, recognizing that definitions of the vaccination rates are not identical across the three prefectures. See also Figures C8 and C9 and Table C2 in Online Appendix C for further statistical checks.

**Table 3:** Regression analysis II: cities/towns/villages level

	Model 8	Model 9
*Demographics*		
% of those aged 65 or older	1.128 *** (0.123)	1.177*** (0.158)
*Governing Parties’ Representation*		
Presence in National Diet		3.582*** (0.493)
*COVID-19*		
Infection Rate		0.037** (0.016)
*Fixed Effects* *(ref: Gifu)*		
Ehime	6.125*** (0.769)	8.091*** (0.320)
Kagawa	6.857*** (0.295)	9.174*** (0.056)
Constant	21.174*** (3.951)	13.554** (5.62)
Observations	79	79
Adjusted R^2^	0.527	0.526
Residual Std Error	8.253 (df = 75)	8.261 (df = 73)
F Static	30.005*** (df = 3; 75)	18.339*** (df = 5; 73)

Notes: Standard errors are clustered for prefectures and are in parenthesis. *p < 0.1, **p < 0.05, ***p < 0.01

Having accounted for the COVID proliferation, albeit limitedly because of data unavailability, the estimation results of Model 9 reaffirm the impact of the political variable, i.e., the presence of (at least one) LDP or Komei Party incumbent representation in the National Diet, on mass vaccination rollouts. Different from Analysis I, the effect of the variable measuring the proportion of those aged 65 or older remains positive and statistically significant, but, as shown, this demographic effect does not take away the impact of our key explanatory variable. The positive estimates associated with the fixed effects for Ehime and Kagawa are expected, given that the reference category, Gifu, was the only prefecture that calculated the vaccination rate by excluding those aged 65 or older. Overall, we take the results reported in Table 3 to be further corroborative evidence pointing to the salience of political determinants in regional vaccination variation in Japan.

## Discussion

Among researchers who utilize survey data and explore factors that influence individual attitudes toward vaccination in Japan, there seems to be an ongoing debate about whether Japanese people are generally hesitant or willing to receive vaccinations [4, 33–35], as well as a continuing discussion regarding whether particular segments of Japan’s population, based on age, gender, and certain professional categories, have distinct vaccination preferences [3, 36–38]. The evidence and interpretations presented in this line of research are far from consistent or conclusive. The inconsistency stems partly from the fact that each study was conducted at different times in relation to various waves of the pandemic, which must have influenced the respective survey results. Even more critically, it must be noted that none of the surveys used in these previous studies was based on a nationally representative sample, but they relied on samples drawn from internet databases. We, as scholars specializing in social surveys and public opinion research, suspect that, at least as for Japan, those who voluntarily register with or who are willing to be called upon as monitors in internet surveys are hardly randomly chosen from the general population. It would thus be difficult to draw a consistent interpretation based on these convenient samples.

In this paper, we have turned our attention rather to the incentives of governing elites who are in the position of being able to influence the vaccination process. Instead of survey data, we took advantage of the aggregate data of the actual number/rate of vaccinations in Japan in our attempt to reveal the political aspects of the contemporary COVID-19 health environment. The findings presented in this paper vindicate the salience of politics, especially the political motivations inherent in democratic institutions in shaping the pattern of vaccine provision and distribution. According to the results of our quantitative analysis, the share/presence of incumbent politicians belonging to the governing parties, the LDP and Komei Party, had a significant effect on the regional variation in mass vaccination rollouts observed in the fall of 2021. We also presented similar results obtained from our analysis for the lower administrative units, namely the cities/towns/villages. In light of the constitutionally guaranteed principles of regional autonomy and self-government under Japan’s democracy, we find it quite remarkable that our analyses identified the parallel patterns of political influence. These findings thus add confidence to our claim that the political dynamics under electoral democracy do constitute a key determinant for regional vaccination variation.

Before closing this section, some limitations of our research must be noted. First, we realize that the electoral interests of incumbent politicians are not the only political factor of importance. Although Japan is not a federal but rather centralized state, the basic administrative capabilities of local governments vary across prefectures as well as their lower-level units. In ordinary times, such capabilities may be measured by the fiscal status and/or the size of local bureaucracy. Indeed, the public services offered, including medical programs and health care subsidies, vary considerably in details from one regional governmental unit to another. Under the COVID-19 health environment, however, what determines each regional government’s ability to handle massive rollouts in an organized and efficient way is not at all clear. In our analysis (for Analysis I), we included the number of doctors as a control variable but, as noted, its effect turned out to be negligible. Given that the doctors are the central providers of vaccinations in each locality, we believe that the inclusion of this variable was essential for our analysis. Nevertheless, the number of qualified doctors only measures the “maximum” mobilizable capacity, and it does not necessarily measure the number of doctors who actually administered vaccinations on a daily basis. We simply had no way of knowing how far the gap was between the maximum number and the actual number for each prefecture. If available, the data on this gap itself, which is likely to vary across local governments, could perhaps serve as a better proxy for their administrative capability under the COVID-19 situation.

Second, studies utilizing aggregate data, like the analysis conducted in this paper, remain vulnerable to criticisms that such studies can only identify correlations, not causalities. We recognize that, despite the highlighted impact associated with the governing parties’ representation, our analysis does not unravel a causal mechanism, or how exactly the share/presence of incumbent politicians belonging to the LDP and Komei Party influenced the progress of vaccination. Did the politicians lobby the national government for the prioritized provision of vaccines to their own constituencies? Or did they urge and pressure each local government to speed up the inoculation? Was there someone, like Prime Minister Suga himself perhaps, orchestrating the entire ordeal? None of these details remains clarified satisfactorily.

Overall, however, our analysis does reveal a glimpse of the political process at work that affected the pattern of vaccine dissemination. Particularly noteworthy in our findings is that the effect of the governing parties’ representation in the national parliament is far more consistent in accounting for the regional variance than that in the prefectural assemblies. In our view, this marked contrast is illustrative. On the one hand, this result may appear surprising; arguably, local politicians are likely to be more attuned to the day-to-day health environment of their constituencies than those representatives in Diet. On the other hand, the apparent influence of the incumbent Diet members becomes not at all puzzling, once their political needs are taken into account. It was not the local politicians but the politicians in the House of Representatives facing the general election within a period of one month or so that were concerned with the public opinion in the constituencies. Evidently, this electoral cycle structured the political dynamics, which we believe in turn yielded the observed regional variation in vaccinations.

Our study focuses on Japan but, elsewhere under democracy, politicians generally care about the chance of reelections. Admittedly, there are vast and often intricate differences across countries in representative institutions, administrative traditions and procedures, and even political cultures. An important task thus remains for future research to explore how these differences affect the pattern of politics involved in vaccinations, and more generally, public health environment related to COVID-19.

## Conclusion

In Japan, Prime Minister Suga ran a vigorous “campaign” for vaccinations from May to September 2021. In light of the anticipated general election, this campaign was also a political campaign. As such, the pattern of vaccine dissemination was bound to be influenced by the motivations of incumbent politicians seeking reelections.

To quote the most famous definition, politics is “the authoritative allocation of values for the society” [39]. For these politicians who were eager to maximize their chances to win elections, vaccines were scarce resources, or “values,” to be allocated, just like the budgets of public works and welfare benefits. In Japan, the power, or “authority” with which to allocate these resources resided in the incumbent politicians who belonged to the LDP and Komei Party, the two parties that have formed the governing coalition for nearly two decades albeit with a short interruption between 2009 and 2012.

COVID-19 has certainly introduced an unusual health environment, but that does not mean that “politics-as-usual” is precluded from governmental decisions and policy directions. As we found in our analyses at both prefectural and sub-prefectural administrative levels, the share/presence of the governing parties’ representation in the national parliament had a positive and statistically significant effect on the region’s vaccination progress. Under democracy, citizens are entitled to expect that equal and fair public health environment is provided for all; the government in turn should meet this expectation and strive as much as possible to promote such an environment. Our paper points to the possibility that competitive nature of democracy may introduce into the making of this environment a significant bias emanating from the composition of incumbent government. We conclude by recommending that citizens should at least be more attentive to this possibility and perhaps engage in public discussion on how to check and remedy such a partisan bias.

## Electronic supplementary material

Below is the link to the electronic supplementary material.

Supplementary Material 1

## Data Availability

The datasets used and/or analyzed during the current study available from the corresponding author on reasonable request.
