# Correction to: Politics of COVID-19 vaccination in Japan: how governing incumbents’ representation affected regional rollout variation

**DOI:** 10.1186/s12889-023-15744-2

**Published:** 2023-04-28

**Authors:** M. Kikuchi, S. Ishihara, M. Kohno

**Affiliations:** 1 Department of Political Science, Washington University in St. Louis, Saint Louis, MO USA; 2 Department of Global Political Economy, Waseda University, Tokyo, Japan; 3 Faculty of Political Science and Economics, Waseda University, Tokyo, Japan

**Correction to**: ***BMC Public Health*23, 515 (2023)**

10.1186/s12889-023-15376-6

Following publication of the original article, the authors identified several typo’s. The correct and incorrect information is listed below, the original publication has been updated. This does not affect the results & conclusions of the publication.

## Incorrect

- We divide not by the population eligible for vaccines but rather by the total population because, as explained below, the exact demographic variable for both eligible and non-eligible for vaccines, i.e., the cohorts aged **12** or younger and aged older than **12**, are not available; we instead control for this group using a proxy variable.
- As for those without vaccine entitlement, we are unable to capture this particular cohort, i.e., those aged **12** or younger, because the Registrar divides age categorization by every five years.
- Positive, if the proliferation is assumed to have an effect of convincing people to get vaccinated sooner. **A rise in COVID-19 spread implies these people (those who died due to COVID-19 infection and those who were infected with COVID-19) will not be available to or lose motivation to receive the vaccines, hence leading to a fall in the number of people who would have received the vaccines.**

## Correct

- We divide not by the population eligible for vaccines but rather by the total population because, as explained below, the exact demographic variable for both eligible and non-eligible for vaccines, i.e., the cohorts aged **11** or younger and aged older than **11** are not available; we instead control for this group using a proxy variable.
- As for those without vaccine entitlement, we are unable to capture this particular cohort, i.e., those aged **11** or younger, because the Registrar divides age categorization by every five years.
- Positive, if the proliferation is assumed to have an effect of convincing people to get vaccinated sooner. **Negative, on the other hand, if a rise in COVID-19 spread implies that those who died due to the infection will no longer be available or those who were infected will lose motivation to receive vaccines, hence leading to a fall in the number of people who would have received the vaccines.**

