# Supplementary material for: Politics of COVID-19 vaccination in Japan: how governing incumbents’ representation affected regional rollout variation
Source: BMC Public Health. 2023 Mar 17;23:515. doi: 10.1186/s12889-023-15376-6 (PMC10021041; doi:10.1186/s12889-023-15376-6)
Supplement: Supplementary file 1 — Supplementary Material 1 [file 12889_2023_15376_MOESM1_ESM.docx]

**Online Appendix:** **Politics of COVID-19 Vaccination in Japan: How Governing Incumbents’ Representation Affected Regional Rollout Variation**

**A: Supplemental figures**


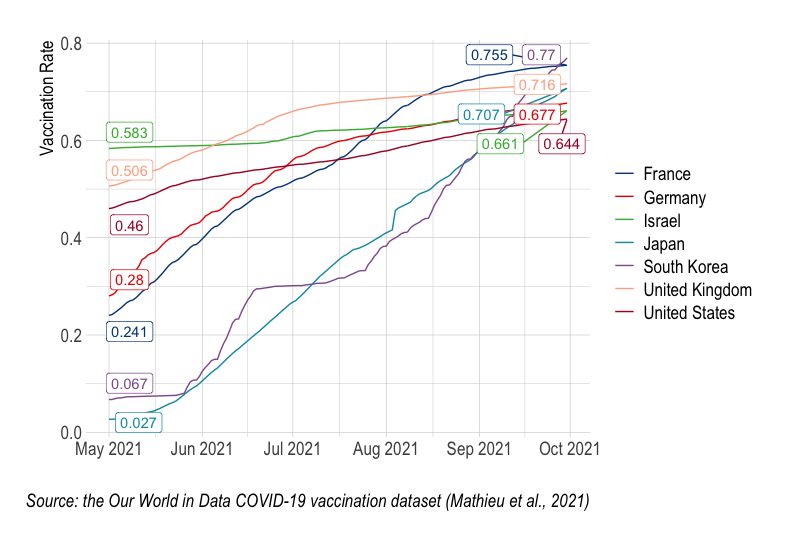
Figure A1 Share of people who received at least one dose of COVID-19 vaccine


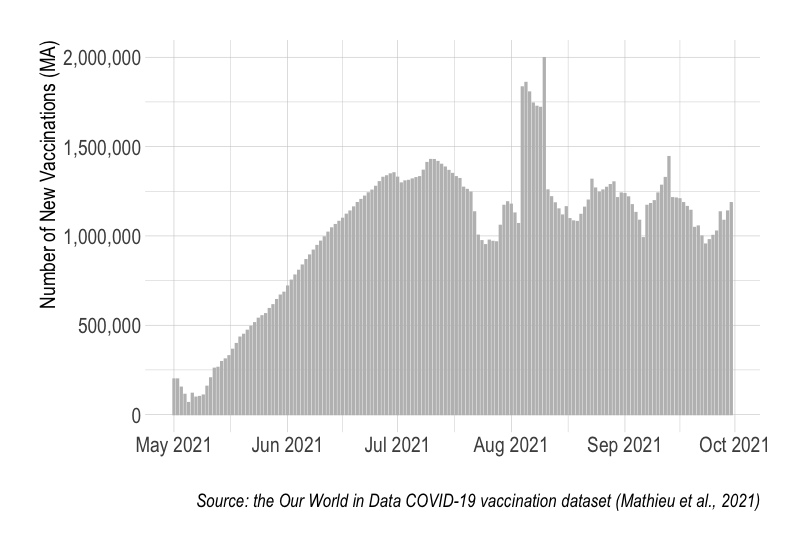


Figure A2 Number of new doses of COVID-19 vaccine per day (7-day smoothed) in Japan

**B. Descriptive Statistics**

Table B1: Descriptive statistics

| Variable | Obs. | Mean | SD | Min | Max |
| --- | --- | --- | --- | --- | --- |
| *Prefectural Level* |  |  |  |  |  |
| Vaccination Rate | 47 | 62.31 | 2.64 | 55.75 | 67.93 |
| % of those aged 65 or older | 47 | 30.37 | 3.1 | 22.35 | 37.23 |
| % of those aged 9 or younger | 47 | 7.7 | 0.77 | 5.89 | 11.06 |
| Share in National Diet | 47 | 77.98 | 25.27 | 0 | 100 |
| Share in Prefectural Assembly | 47 | 65.23 | 12.75 | 31.25 | 84.21 |
| Infection Rate | 47 | 87.55 | 64.93 | 19.18 | 339.39 |
| Death Rate | 47 | 0.89 | 0.7 | 0.04 | 3.34 |
| # of doctors (per 100,000) | 47 | 264.3 | 42.55 | 176.4 | 346.7 |
| *Cities/Towns/Villages Level* |  |  |  |  |  |
| Vaccination Rate | 79 | 62.75 | 12 | 35.8 | 87.2 |
| % those aged 65 or older | 79 | 34.19 | 6.41 | 21.24 | 48.6 |
| Presence in National Diet | 79 | 0.89 | 0.32 | 0 | 1 |
| Infection Rate | 79 | 48.01 | 31.8 | 0 | 147.12 |

**C. Validity for adopting OLS model**

In this section, we provide additional analyses to validate the adoption of OLS model in our analysis. First, we examine heteroskedasticity for all OLS models in the main text by focusing on the relationships between the residuals and fitted values. Figures C1 through C9 display the results, suggesting that the residuals are distributed randomly around the zero line. As we notice a possible outlier from Figures C1 to C7, i.e., Okinawa prefecture, we estimated without Okinawa for robustness checks. Because these additional analyses do not alter any of our significant results, we do not report here. Overall, these patterns confirm the validity of employing the linear model to analyze our dataset.

In addition, we investigate the potential risks of multicollinearity among the independent variables in the models. To address this issue, we report the Variance Inflation Factor (VIF) in Tables C1 and C2. Table C1 shows that when Infection Rates and Death Rates are incorporated in the model simultaneously, the VIF of Infection Rates exceeds 5 (Model 4 and Model 7), a threshold frequently used for indicating risks for multicollinearity (the other threshold sometimes used is 10). However, the estimates of our interests, Share in National Diet, remain stable across the models. Thus, we can confirm that results of the OLS regressions are not influenced by a potential multicollinearity problem.


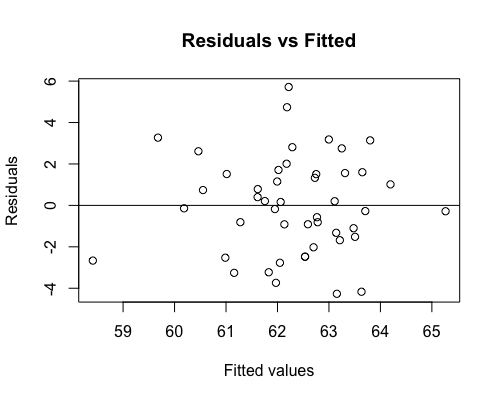


Figure C1: Residual versus fits plot for Model 1


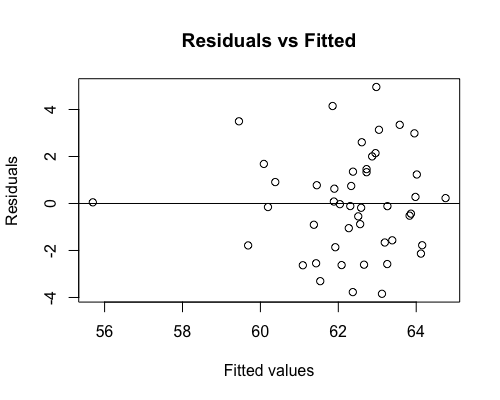


Figure C2: Residual versus fits plot for Model 2


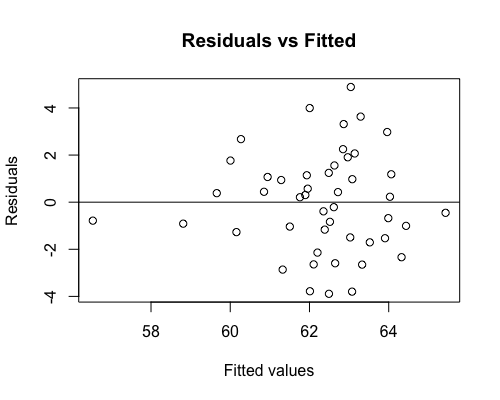


Figure C3: Residual versus fits plot for Model 3


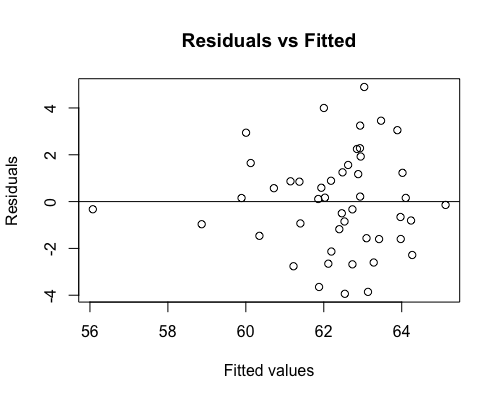


Figure C4: Residual versus fits plot for Model 4


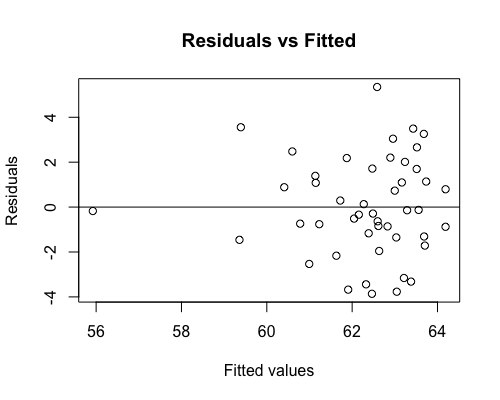


Figure C5: Residual versus fits plot for Model 5


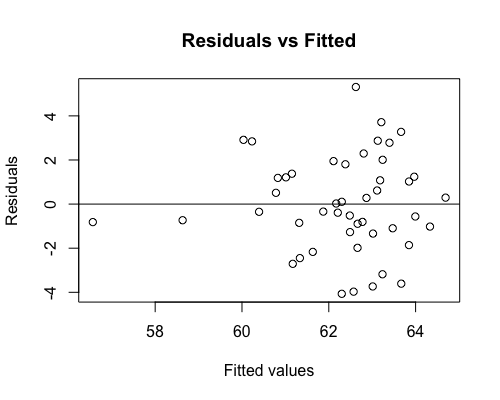


Figure C6: Residual versus fits plot for Model 6


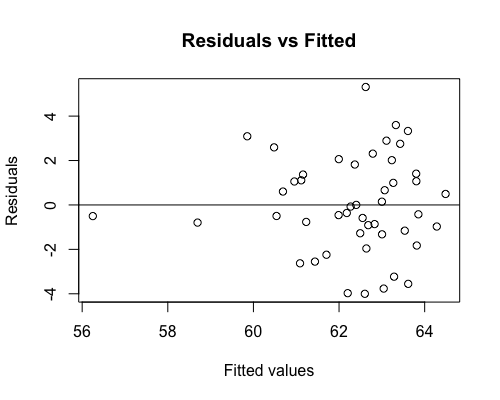


Figure C7: Residual versus fits plot for Model 7


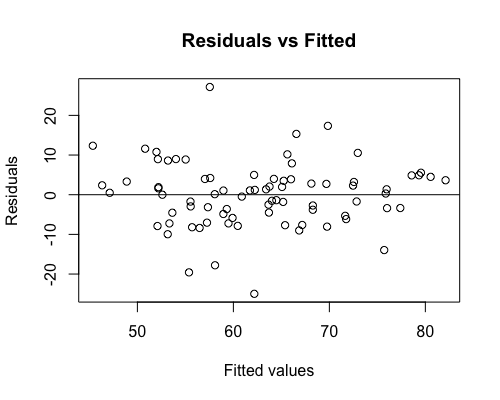


Figure C8: Residual versus fits plot for Model 8


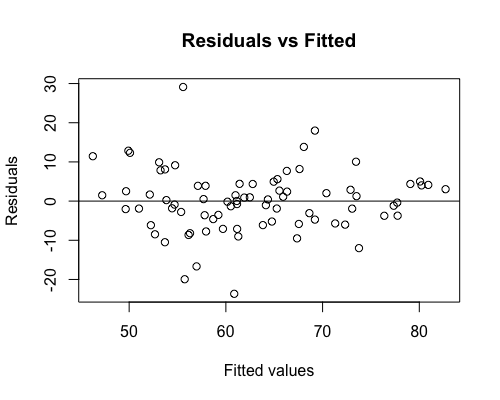


Figure C9: Residual versus fits plot for Model 9

Table C1. VIF for Prefectural Level Models

| **Variable** | **Model 1** | **Model 2** | **Model 3** | **Model 4** | **Model 5** | **Model 6** | **Model 7** |
| --- | --- | --- | --- | --- | --- | --- | --- |
| % of those aged 65 or older | 1.63 | 4.7 | 2.97 | 4.71 | 4.65 | 3.02 | 4.66 |
| % of those aged 9 or younger | 1.63 | 1.85 | 2.04 | 2.13 | 1.93 | 2.04 | 2.2 |
| Share in National Diet |  | 1.08 | 1.06 | 1.08 |  |  |  |
| Share in Prefectural Assembly |  |  |  |  | 1.39 | 1.28 | 1.39 |
| Infection Rate |  | 3.7 |  | 6.81 | 4.25 |  | 7.21 |
| Death Rate |  |  | 1.7 | 3.12 |  | 1.84 | 3.12 |
| # of doctors (per 100,000) |  | 1.26 | 1.23 | 1.26 | 1.23 | 1.21 | 1.23 |

Table C2. VIF for Cities/Towns/Villages Level Models

| **Variable** | **Model 8** | **Model 9** |
| --- | --- | --- |
| % of those aged 65 or older | 1.2 | 1.68 |
| Presence in National Diet |  | 1.28 |
| Infection Rate |  | 2.38 |
| Ehime | 1.32 | 1.92 |
| Kagawa | 1.13 | 1.71 |

**D: Robustness Check**

Some critical readers might be concerned that the shortage of nurses, in addition to that of doctors, confounds with the relationships between vaccination rates and the share of the governing coalition. To mitigate this concern, we collected an additional variable, # of nurses (per 100,000) and decided to run additional analyses for robustness check. A potential multicollinearity problem was found, however, by adding the variable in our model in that this variable correlates with % of those aged 65 or older. We thus choose not to report in the main text but present the results of OLS estimations here. Models 1-7 in Table C1 indicate that the effect of our main independent variable, Share in National Diet, remains almost the same consistently. These findings assure that the administrative capacity of vaccination rollouts is sufficiently held constant in our original estimations.

Table D1: Regression Analysis with # of nurses (per 100,000)

| Variable | Model C1 | Model C2 | Model C3 | Model C4 | Model C5 | Model C6 | Model C7 |
| --- | --- | --- | --- | --- | --- | --- | --- |
| *Demographics* |  | | | | | | |
| % of those aged 65 or older | 0.339 ** (0.145) | 0.049 (0.370) | 0.021 (0.362) | -0.011 (0.375) | 0.152 (0.374) | 0.147 (0.364) | 0.113 (0.381) |
|  |  |  |  |  |  |  |  |
| % of those aged 9 or younger | -0.349 (0.584) | -0.307 (0.911) | -0.806 (0.956) | -0.704 (1.004) | -0.350 (0.938) | -0.715 (0.978) | -0.617 (1.030) |
|  |  |  |  |  |  |  |  |
| *Governing Parties’ Representation* |  | | | | | | |
| Share in National Diet |  | 0.029** (0.014) | 0.032** (0.014) | 0.031** (0.014) |  |  |  |
|  |  |  |  |  |  |  |  |
| Share in Prefectural Assembly |  |  |  |  | 0.05 (0.032) | 0.052* (0.030) | 0.049 (0.032) |
|  |  |  |  |  |  |  |  |
| *COVID-19 Rates (per 10,000)* |  | | | | | | |
| Infection Rate |  | -0.014 (0.01) |  | -0.005 (0.014) | -0.011 (0.011) |  | -0.005 (0.014) |
|  |  |  |  |  |  |  |  |
| Death Rate |  |  | -1.070* (0.634) | -0.841 (0.885) |  | -0.790 (0.678) | -0.589 (0.902) |
|  |  |  |  |  |  |  |  |
| # of doctors (per 100,000) |  | 0.003 (0.014) | -0.0002 (0.14) | 0.001 (0.014) | 0.0010 (0.014) | 0.008 (0.013) | 0.009 (0.014) |
|  |  |  |  |  |  |  |  |
|  |  |  |  |  |  |  |  |
| # of nurses (per 100,000) |  | 0.00003  (0.005) | 0.002  (0.005) | 0.001  (0.005) | -0.002  (0.005) | -0.001  (0.005) | -0.002  (0.005) |
| Constant | 54.684*** (8.012) | 61.218*** (14.895) | 64.567*** (15.232) | 65.156*** (15.479) | 58.030*** (15.222) | 60.004*** (15.565) | 60.753*** (15.890) |
| Observations | 47 | 47 | 47 | 47 | 47 | 47 | 47 |
| Adjusted R^2^ | 0.184 | 0.250 | 0.265 | 0.249 | 0.220 | 0.226 | 0.209 |
| F Static | 6.185*** (df = 2; 44) | 3.560*** (df = 6; 40) | 3.759*** (df = 6; 40) | 3.173*** (df = 7; 39) | 3.165*** (df = 6; 40) | 3.242*** (df = 6; 40) | 2.735** (df = 7; 39) |
|  |  |  |  |  |  |  |  |

Notes: Standard errors are in parenthesis. *p<0.1, **p<0.05, ***p<0.01

**References**

Ministry of Health, Labor and Welfare. Report on Public Health Administration and Services for the Year 2018. https://www.mhlw.go.jp/toukei/saikin/hw/ishi/18/index.html. Accessed 24 November 2022.
